# Transport via Macropinocytic Vesicles Is Crucial for Productive Infection with Bombyx Mori Nucleopolyhedrovirus

**DOI:** 10.3390/v11070668

**Published:** 2019-07-20

**Authors:** Jinshan Huang, Chenya Li, Xudong Tang, Lin Liu, Wenbin Nan, Xingjia Shen, Bifang Hao

**Affiliations:** 1 Jiangsu Key Laboratory of Sericultural Biology and Biotechnology, School of Biotechnology, Jiangsu University of Science and Technology, Zhenjiang 212018, Jiangsu, China; 2 Key Laboratory of Genetic Improvement of Sericulture in the Ministry of Agriculture, Sericultural Research Institute, Chinese Academy of Agricultural Science, Zhenjiang 212018, Jiangsu, China

**Keywords:** baculovirus, fusion protein, infection, membrane fusion

## Abstract

Bombyx mori nucleopolyhedrovirus (BmNPV) is a serious viral pathogen in the sericulture industry and enters host cells via macropinocytic endocytosis; however, the current understanding of the BmNPV entry mechanism remains limited. To confirm whether direct membrane fusion (DMF) results in productive BmNPV infection, DMF infectivity induced by low pH during BmNPV infection was investigated, and the infectious viral particle was traced using an eGFP-labeled virion. We found that BmNPV infection efficiently induced fluid uptake, which allowed BmNPV to bypass the cell membrane barrier via macropinocytosis. However, DMF induced by a low pH abolished the infection. While low pH is an essential condition for membrane fusion triggering, it is not sufficient for productive BmNPV infection, and DMF results in failure to transport the nucleocapsid into the nucleus. These results indicate that transport via macropinocytic vesicles facilitates BmNPV entry into the nucleus and contribute to our understanding of the BmNPV entry mechanism.

## 1. Introduction

Baculoviruses are rod-shaped, enveloped DNA viruses that are 230–385 nm in length and 40–60 nm in diameter that infect insects of the orders Lepidoptera, Diptera, and Hymenoptera [1]. Autographa californica multiple nucleopolyhedrovirus (AcMNPV) and Bombyx mori nucleopolyhedrovirus (BmNPV) are Group I alphabaculoviruses that have acquired the GP64 membrane fusion protein during evolution [2]. Although the BmNPV genome shares over 90% identity with that of AcMNPV [3], these viruses have very different host ranges; AcMNPV infects a much more diverse set of insects and insect cell lines, while the natural host of BmNPV is limited to the silkworm [1].

AcMNPV enters the host cells by clathrin-mediated endocytosis (CME), thus bypassing the plasma membrane [4]. GP64 mediates viral membrane fusion with the membranes of endocytic vacuoles, a process triggered by the low pH in the endosome lumen [5]. Then, nucleocapsids are released into the cytosol and transported into the nucleus for replication [6]. However, when endosomal CME maturation is prevented by incubation with ammonia chloride, DMF is induced by a low pH; subsequently, naked nucleocapsids are released into the cytoplasm from early endosomes, and nucleocapsids are transported into the nucleus to initialize infection [4], indicating that virion transport in endosomes is not essential for productive AcMNPV infection. Furthermore, CME, DMF, and macropinocytosis are employed by AcMNPV to enter mammalian cells [7].

Our previous study showed that BmNPV enters host cells by macropinocytosis [8]. Macropinocytic endocytosis has been reported to be used by some large viruses, such as vaccinia virus, to enter host cells [9]. Furthermore, DMF was used as an alternative mechanism to expand the range of host cells for vaccinia virus [10,11]. Thus, determining whether DMF results in a productive BmNPV infection is interesting. In the present study, we further characterized the BmNPV entry mechanism, and we report for the first time that macropinocytic transport, but not DMF, is critical for a productive BmNPV infection.

## 2. Materials and Methods 

### 2.1. Cells and Viruses

The BmN [8] and Bm5 [12] cell lines were cultured at 27 °C in TC-100 insect medium (Applichem, Darmstadt, Germany) supplemented with 10% fetal bovine serum (Gibco-BRL, MD, USA) using standard techniques. The Sf9 cell line (Thermo Fisher Scientific, MA, USA) was cultured at 27 °C in SF900IISFM insect medium (Thermo Fisher Scientific, MA, USA) using standard techniques. BmBac-GFP [8] and AcBac-GFP [4] were constructed previously. In brief, egfp gene was inserted under the p10 promoter to generate recombinant plasmid pFastBac-egfp. The recombinant virus BmBac-GFP and AcBac-GFP were generated by transforming DH10Bac™ *Escherichia coli (*Thermo Fisher Scientific, MA, USA) and transfecting the indicated bacmids into BmN or Sf9 cells according to the manufacturer’s instructions (Bac-to-Bac baculovirus expression system, Thermo Fisher Scientific, MA, USA), and viral titers were determined by the endpoint dilution assay.

### 2.2. Construction of Transient Expression Vector and Immunofluorescence Assay

The BmNPV *gp64* gene was amplified with primers 5′ CGCGAATTCGACAGATATTTAAAT AAACCAAAC-3′ and 5′ GCGTCTAGATTAATATTGTCTACTATTACGGTT-3′, and the products were digested and cloned into the pIZ-V5 vector by *Bam*H I and *Hin*d III to construct the transient expression vector pIZ-V5-gp64. BmN cells were seeded on 22-mm coverslips placed in 6-well plates and maintained at 27 °C overnight. The cells were then transfected with 0.5 μg of pIZ-V5-gp64 and cultured at 27 °C for 72 h. Transfected cells were fixed with 4% paraformaldehyde in PBS (pH 7.4) at room temperature and washed three times with PBS. The fixed cells were then blocked in PBS containing 5% bovine serum albumin for 30 min and incubated for two hours with an anti-GP64 antibody diluted to 1:2000 in PBS. The cells were then washed three times with PBST and reacted for 1 h with an AlexaFluor 488-conjugated goat anti-rabbit IgG antibody (Thermo Fisher Scientific, USA) diluted to 1:2000 in PBS. The cells were then washed three times with PBST and incubated with Hoechst33258 (final concentration 1 μg/mL, MS, Sigma-Aldrich) in PBS for 30 min. The washing process with PBST was repeated three times, and the cells were imaged using a confocal laser scanning microscope (Leica SP8).

### 2.3. Syncytium Formation Assay

The membrane fusion activities of BmNPV GP64 were determined using a syncytium formation assay as previously described [13]. BmN cells transfected with 0.5 μg of pIZ-V5-gp64 DNA were then exposed to media with average endosomal pH values of 6.2, 5.5, 5.0, and 4.5 for 5 min at 72 h post-transfection (p.t.), followed by incubation in the normal-pH medium for 4 h. DNA was stained with Hoechst33258 (final concentration 1 μg/mL, Sigma), and syncytia were microscopically imaged. A syncytium was defined as a group or mass of fused cells containing at least 5 nuclei. Relative levels of fusion activity were determined by dividing the total number of nuclei in syncytia by the total number of nuclei in the same field and normalizing to corresponding data from cells expressing equivalent levels of GP64 localized to the cell surface. Ten fields were selected randomly for the statistical analysis of syncytia.

### 2.4. Reversible Conformational Change Assay

A one hundred microliter BmBac-GFP stock solution (titer = 2.86 × 10^6^ TCID_50_) at pH 6.2 was adjusted to pH 4.5 with PBS (50 µl, pH 1.7). The low-pH-adjusted virus was incubated for 30 min at room temperature with a rotator and then returned to pH 6.2 by the addition of PBS (60 µl, pH 12.3). The same treatment was conducted with PBS (pH 6.2) as the control. The titers of the two viruses were then determined by the endpoint dilution assay. All of the experiments were repeated three times, and standard deviations and Student’s t-test results were calculated by Microsoft Excel 2016.

### 2.5. Fluid Uptake Assay

BmN or Bm5 cells were seeded in confocal dishes and cultured overnight. To evaluate fluid uptake, a 30 μL mixture of lucifer yellow (LY, 1 mg/mL) and BmBac-GFP (multiplicity of infection [MOI] of 10 TCID_50_) was added to 500 μl of medium and incubated for 30 min to initialize the infection. The unbound virus and medium were removed, and the cells were washed twice with PBS. The DNA was then stained with Hoechst33258. The fluid uptake was observed and recorded by a Leica SP8 laser confocal microscope at 30 min post-infection (p.i.). Thirty cells were selected randomly for average macropinosomes per cell statistics. Student t-test results were calculated by Microsoft Excel 2016.

### 2.6. Ammonium Chloride Treatment and DMF Inducement

BmN cells were seeded in a 24-well culture plate overnight, incubated with ammonium chloride (25 mM, Sangon, Shanghai, China) and prechilled for 40 min at 4 °C; BmBac-GFP (MOI of 5 TCID_50_/cell) was then added to the plate to initiate the infection at 4 °C for 60 min. Unbound virus was removed, and the cells were exposed to pH 4.5 medium for 5 min at 27 °C to induce DMF. The low-pH medium was discarded, and the cells were then cultured in normal-pH (6.2) medium for 48 h. Fluorescence expression was recorded by an Olympus IX43 microscope.

### 2.7. Flow Cytometry Analysis

The infected cells were washed twice with PBS and then resuspended in 1 ml of PBS for the flow cytometry (BD) assay. Ten thousand events were acquired in the gate for each treatment, and the experiments were repeated three times; standard deviations and Student’s t-test results were calculated by Microsoft Excel 2016.

### 2.8. DMF Comparison between AcBac-GFP and BmBac-GFP Infection

BmN, Bm5, and Sf9 cells were seeded in 24-well culture plates overnight, pretreated with ammonium chloride (25 mM) for 40 min and then infected with BmBac-GFP (MOI of 5 TCID _50_/cell for BmN and Bm5 cells infection; and MOI of 30 TCID_50_/cell for Sf9 cells) at 4 °C for 60 min or AcBac-GFP (MOI of 5 TCID_50_/cell). The unbound viruses were removed, and the cells were incubated with a low-pH medium for 5 min at 27 °C. The low-pH medium was discarded, and the cells were cultured overnight, fluorescence expression was recorded by an Olympus IX43 microscope.

### 2.9. Construction of Recombinant Labeled Virus

*Egfp* gene without the stop codon was amplified with the primers 5′-CGGGGATCCATGGTGAGCAAGGGCGAGG-3′ (*Bam*H Ι site underlined) and 5′-GCGGTCGACCTTGTACAGCTCGTCCATGC-3′ (*Sal* I site underlined) from pEGFP-N1 (Clontech, TaKaRa, Japan). The products were inserted under the polyhedrin promoter of pFastBacDual plasmid by *Bam*H Ι and *Sal* I to generate pFBD-egfp. The BmNPV *vp39* gene was amplified with the primers 5′-GGCGCGGCCGCATGGCGCTAATGCCCGTGG-3′ (*Not* Ι site underlined) and 5′-GCGAAGCTTTTAGGCGGCTACACCTCCG-3′ (*Hin*d Ⅲ site underlined) from BmBacJS13. The products were digested by *Not* Ι and *Hin*d Ⅲ and cloned into pFBD-egfp to generate pFBD-egfp-vp39, in which *vp39* gene was fused to C-terminus of *egfp*. The recombinant bacmid BmBac-GFP-VP39 was generated by Tn7-based transposition of the egfp-vp39 into BmBacJS13. BmBac-GFP-VP39 was transfected into and used to infect BmN cells to produce budded virus (BV) with GFP-labeled VP39 in nucleocapsid. Five milliliters of BVs were ultracentrifuged, and the pellet was collected for Western blotting with an anti-VP39 antibody.

### 2.10. Tracing of Viral Particles during Infection

BmN cells were seeded in confocal dishes overnight culture. Cells were pretreated with ammonium chloride (25 mM) for 40 min and were then infected with BmBac-GFP-VP39 (MOI, 50 TCID_50_/cell) for 1 h at 4 °C to initiate macropinocytosis entry. The BVs were then removed and exposed to pH 4.5 medium for 5 min to interrupt the macropinocytic transportation by induced DMF. The low-pH medium was then removed, and cells were incubated with a normal medium for confocal microscopic observation. The control cells were treated and incubated with pH 6.2 media to continue the macropinosome maturation. The DNA was stained with Hoechst33258.

## 3. Results

### 3.1. Membrane Fusion Induced by BmNPV GP64 is Triggered by Low pH

**Figure 1:**
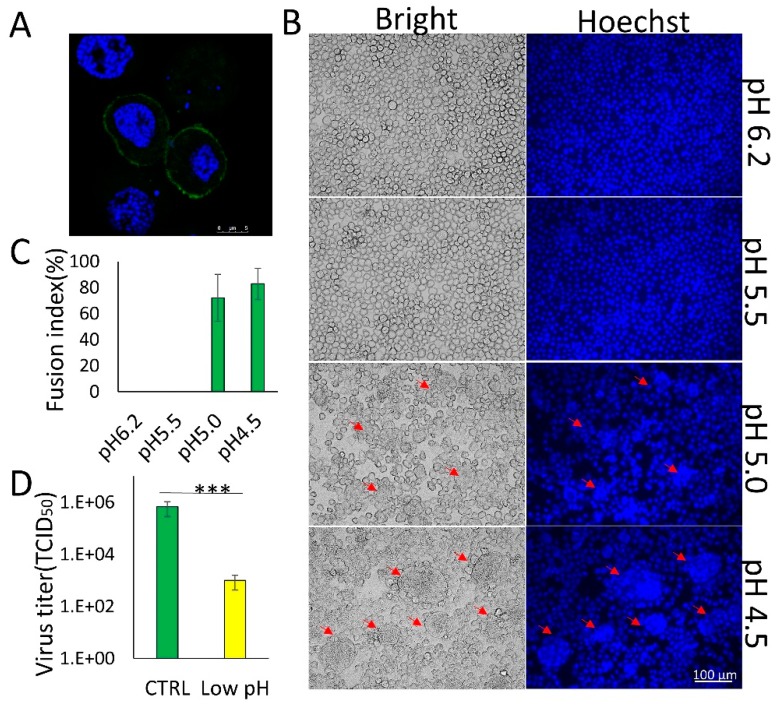
BmNPV is an acid-activated virus with a low triggering pH for GP64 conformational change. (**A**) Localization of BmNPV GP64 by immunofluorescence analysis with GP64 antibody and FITC-conjugated rabbit anti-mouse antibodies. Scale bar, 5 μm. (**B**) Syncytium formation assay. BmN cells were transfected with pIZ-V5-gp64 DNA and exposed to low-pH (4.5–6.2) medium to induce syncytium formation at 72 h p.t. The arrows show the syncytia induced by the low pH. (**C**) Comparison of the fusion index of different low-pH treatments. Error bars represent standard deviations. (**D**) Reversible conformational change in BV of BmNPV. BmBac-GFP was exposed to low pH and then returned to normal pH, and titers were then determined by endpoint dilution assay on BmN cells. *** *p* < 0.001.

The pH dependence of membrane fusion varies among enveloped viruses [14] and can be correlated with the endosomal site of fusion [15]. AcMNPV requires a pH of 5.5 or lower to trigger membrane fusion with host cells [13], whereas the pH threshold for low-pH-triggered membrane fusion in BmNPV N 9 strain is lower pH than that of AcMNPV [16], however, this trigger pH value outranges the endosomal pH. To further explore the pH value necessary to trigger membrane fusion, BmNPV Shaanxi strain GP64 was transiently expressed in BmN cells. The exogenously expressed GP64 was localized to the plasma membrane of the transfected cells (Figure 1A). When the cells were exposed to low-pH medium (pH 5.0 or pH 4.5), syncytia were formed (Figure 1B, arrows), which were more obvious when cells were exposed to a pH of 4.5 (Figure 1C). However, syncytia were not formed upon exposure to a medium with a pH of 6.2 or 5.5. Compared to AcMNPV, BmNPV requires an even lower pH (≤ 5.0) to trigger a conformational change in GP64. When a BmBac-GFP BV [8] was pre-exposed to a low pH and then subjected to a normal-pH environment, the BV exhibited greatly reduced infectivity than the control, only 1.5‰ of its activity was retained (Figure 1D). These results indicate that BmNPV GP64 can trigger plasma membrane fusion under low-pH conditions. However, the conformational change in GP64 is irreversible after exposure to a low pH, and virus infectivity is greatly reduced.

### 3.2. Virus-Activated Fluid Uptake Mediates BmNPV Entry into Host Cells

**Figure 2:**
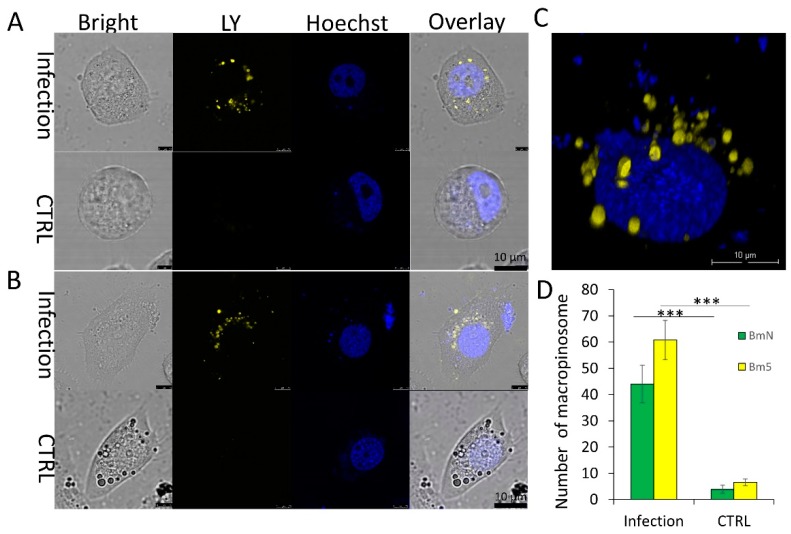
Fluid uptake assay of BV entry into host cells. The fluid uptake levels in BmN (**A**) and Bm5 (**B**) cells activated by BmBac-GFP infection at 30 min p.i. Scale bar, 10 μm. (**C**) Comparison of macropinosome numbers in BmN and Bm5 cells. Error bars represent standard deviations. ***p < 0.001. (**D**) Reconstruction of 3D scanning for macropinosomes in BmN cells. Scale bar, 10 μm.

Macropinocytosis is a transient, actin-dependent, endocytic process that leads to internalization of fluid through membrane ruffling. Thus, fluid uptake is a key criterion for macropinocytic endocytosis [9]. To further determine whether macropinocytosis is employed by BmNPV for entry into host cells, we assessed the intensity of ruffling caused by BmNPV infection. LY, a marker used to label fluid during macropinocytosis, was added to the medium to form many labeled macropinosomes after engulfment by BmN and Bm5 cells (Figure 2A,B). Calculation of the average number of macropinosomes per cell showed increases of 11.5- and 9.8-fold in infected BmN and Bm5 cells, respectively, compared to that in uninfected cells (Figure 2C). LY-labeled macropinosomes were internalized to the region around the nucleus (30 min p.i.) (Figure 2D). In addition, the engulfed macropinosomes were retained in the cytoplasm within 60 min after infection. Furthermore, the mean LY intensity in an infected cell stabilized (approximately 113.5 ± 1.12 relative fluorescence intensity units), indicating that macropinosomes may not undergo recycling but may enter the degradation cycle by fusion with lysosomes to generate a transient endolysosome.

### 3.3. DMF Results in a Productive Infection for AcMNPV but an Abortive Infection for BmNPV

**Figure 3:**
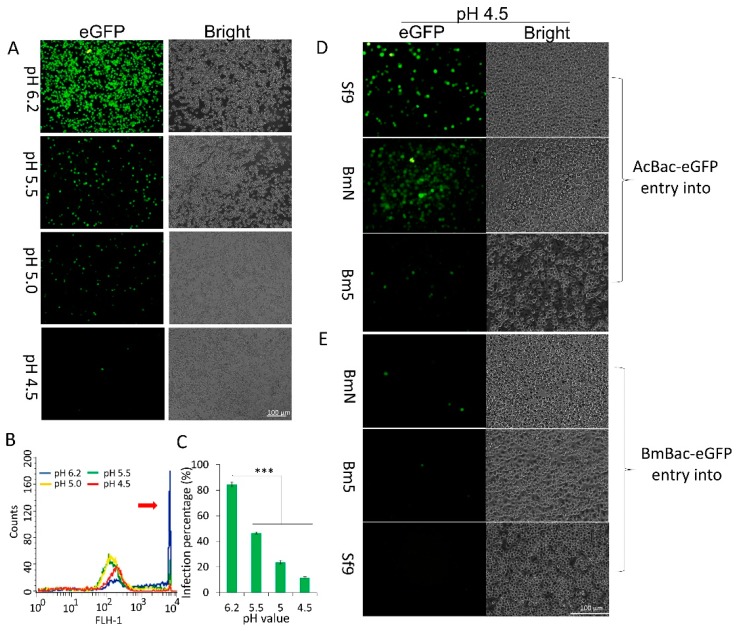
BmNPV productive infection abolished by low-pH-induced direct membrane fusion (DMF). (**A**) Representative images of fluorescence expression in BmN cells induced by DMF. Scale bar, 100 µm. (**B**) Fluorescence detection of the cells treated by DMF via flow cytometry. The red arrow represents fluorescent cell peaks. (**C**) Histogram representing the percentage of infected BmN cells induced with media with different pH values. ***p < 0.001. Representative images of fluorescence expression in Sf9, BmN, and Bm5 cells infected by AcBac-GFP (**D**) and BmBac-GFP (**E**). Scale bar, 100 µm.

BmN cells were preincubated with ammonium chloride, an inhibitor that prevents endosome maturation by elevating the pH of the late endosome [17]. Subsequently, the cells were infected with BmBac-GFP at 4 °C for 1 h, after which they were exposed to media with different pH values for 5 min to trigger DMF in immature macropinosomes and were then cultured in pH 6.2 medium. The infection rate was measured at 48 h p.i. The number of infected cells decreased with the pH value, and only single fluorescent cells were detected after treatment with pH 4.5 medium (Figure 3A). Flow cytometric analysis revealed that the number of cells with strong fluorescence decreased rapidly (Figure 3B, red arrow), and a significant difference was detected between cells exposed to normal treatment (pH 6.2) and those exposed to the three low-pH values (Figure 3C).

Low-pH-induced DMF has been reported to efficiently mediate AcMNPV entry into the host and mammalian cells [4]. The infectivity of AcBac-GFP and BmBac-GFP were compared in the host or non-host cells after low-pH-induced DMF. The results were similar to those of Dong et al. [4], the infectivity of AcBac-GFP after pH 4.5 treatment were comparable to those after pH 6.2 treatment in both host Sf9 cells and non-host cells (BmN and Bm5) (Figure 3D). In contrast, after low-pH treatment, BmBac-GFP exhibited greatly reduced infectivity in host cells (BmN and Bm5) and in non-host Sf9 cells at a high MOI (Figure 3E). Taken together, these results revealed that exposure to a low pH resulted in an abortive infection by BmNPV but not by AcMNPV.

### 3.4. DMF-Mediated Abortive Infection of BmNPV Results from a Failure of Nucleocapsid Transport to the Nucleus

**Figure 4:**
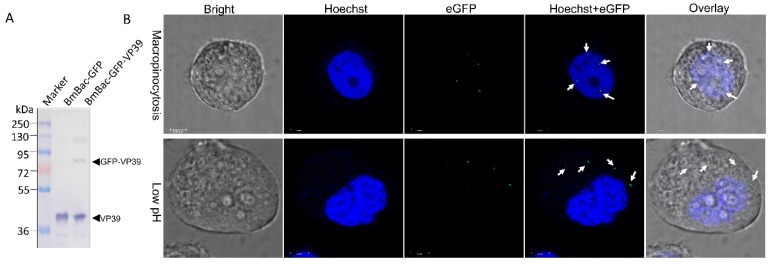
Viral particle tracing analysis on macropinocytosis and low-pH-induced DMF. (**A**) Western blot analysis of recombinant virus BmBac-GFP-VP39. BVs were collected by ultracentrifugation and were subjected to Western blotting. The arrows indicate VP39 (lower arrow) and GFP-VP39 (upper arrow). (**B**) Localization assay of eGFP-labeled nucleocapsids in the macropinocytosis and DMF treatment (2 h p.i.). The arrows show the nucleocapsids in the nucleus (upper panel) or in the cytoplasm (lower panel). Scale bar, 5 μm.

To further explore the mechanism of the DMF-mediated abortive infection of BmNPV in host cells, the nucleocapsid transport during infection was examined. A recombinant virus containing eGFP fused to VP39 (BmBac-GFP-VP39) was constructed (Figure 4A) and applied for viral particle tracing. Under the macropinocytosis condition, eGFP-labeled nucleocapsids were localized in the cytoplasm at the beginning of the infection and were ultimately transported into the nucleus at 2 h p.i. (Figure 4B, arrows in upper panel). When the infected cells were exposed to a low pH in the DMF condition, the labeled nucleocapsid was distributed in only the cytoplasm over time (Figure 4B, arrows in lower panel), it was difficult to observe nucleocapsids in the nucleus. These results demonstrate that DMF results in a failure to import the BmNPV nucleocapsid into the nucleus, resulting in an abortive infection.

## 4. Discussion

Enveloped viruses deliver their genomes into the cytoplasm by fusing with the endosomal or plasma membrane, which is triggered by the low pH of the endosome [18], and most DNA viruses continue their journey to the nucleus to initiate a productive infection [19]. Here, we showed that for BmNPV, a pH 5 or lower was essential for a conformational change in GP64, and this change was irreversible after viruses were exposed to a low pH, indicating that BmNPV is an acid-dependent virus. Together with another study, a lower trigger pH to induce a conformational change in BmNPV N9 GP64 [16], we confirmed that a more acidic environment is required for BmNPV than for AcMNPV to induce a conformational change in GP64 [13]. This difference was similar to that observed for the HA subtype of influenza virus, which can vary by ∼0.7 pH units among different strains [20], which may have been caused by mutations of key amino acids during evolution [16].

According to the pH threshold for acid activation in endosomes, viruses are classified as early-penetrating viruses (E-PVs) and late-penetrating viruses (L-PVs) [21]. E-PVs generally fuse to early endosomes (EE) and are triggered by a pH of 6.5–6.0. Examples of E-PVs include Semliki Forest virus [15] and Vesicular stomatitis virus [22]. L-PVs generally fuse to late endosomes or lysosomes and are triggered by pH of 6.0–5.0. L-PVs include most strains of influenza [21]. Hence, AcMNPV and BmNPV are considered L-PVs based on their pH threshold. However, a recent study indicated that AcMNPV penetration occurred in EEs [23]. In addition, the nucleocapsids released into the cytoplasm by DMF can be transported into the nucleus in AcMNPV infection [4,6], which is similar to that of Semliki forest virus and Vesicular stomatitis virus [24], a low pH is sufficient to mediate virus infection; thus, AcMNPV can be considered an E-PV.

Previously, we used several inhibitors of the key factors involved in macropinocytic endocytosis to validate the entry mechanism of BmNPV [8]. Fluid uptake is a key criterion for macropinocytosis [9]. BmNPV infection significantly activated fluid uptake, and fluid uptake analysis further verified the BmNPV entry pathway, as the macropinosome number increased by 5- to 10-fold in macropinocytic endocytosis [9]. In addition, the most effective trigger pH for syncytia formation mediated by GP64 is a low lysosomal pH value [25]. It is known that mature macropinosomes will fuse with lysosomes after macropinocytosis [25], therefore, we suggested that BmNPV was an L-PV. 

L-PVs are particularly sensitive to perturbations that interfere with the maturation of endosomes [21]. DMF treatment completely disrupts the maturation of macropinosomes, and BmNPV nucleocapsids were released into the cytoplasm from macropinosomes. This release resulted in greatly reduced BmNPV infectivity due to a failure to transport the nucleocapsid into the host cell’s nucleus, which is also the cause of the failure of BmNPV infection in non-host cells [26]. These results indicate that the nuclear membrane is the key barrier for not only productive infection by BmNPV in non-host cells but also for infection of host cells; however, macropinocytic endocytosis was adapted by BmNPV, as it can efficiently bypass these two barriers. AcMNPV nucleocapsids can be transported into the nucleus through a nuclear pore [27] via interaction with actin/P78–83 [4,6], while this mechanism may not involve the entry of BmNPV into host and non-host cells, this may result from the absence of some AcMNPV genes in BmNPV genome [3], such as pcna, host cell-specific factor 1, ac134, it explains why low-pH triggering is not sufficient for BmNPV infection. Hence, there must be another mechanism for BmNPV nuclear entry.

L-PVs must be sorted into a degradative pathway by fusion with endolysosomes/lysosomes [21]. Ebola virus enters host cells by macropinocytosis [28], a low-pH trigger is essential but not sufficient for infection, as Ebola GP requires endosomal proteolysis for priming in lysosomes [29]. We found engulfed macropinosomes retained in the cytoplasm in this study, which implies that degradation may be essential for BmNPV infection. The main difference between DMF and macropinocytosis treatment is that macropinosome maturation in lysosomes is lost in DMF. Lysosomes are active degradation organelles [30] and are essential for productive infection by L-PVs [21], though most internalized virions are degraded [31]. Furthermore, lysed macropinosome components are required for penetration and infection by some viruses, and this process involves other endocytosis pathways [32]. We found that chemically induced macropinocytosis can mediate productive infection by BmNPV in Sf9 cells (China patent ZL201610384217.6), hence, macropinocytic endocytosis is an effective entry mechanism for BmNPV. However, macropinocytosis is still an incompletely characterized pathway [9], and the mechanisms of BmNPV entry and its degradation require further investigation.
